# Cardiovascular Protective Properties of GLP-1 Receptor Agonists: More than Just Diabetic and Weight Loss Drugs

**DOI:** 10.3390/jcm13164674

**Published:** 2024-08-09

**Authors:** Richard Le, Mau T. Nguyen, Momina A. Allahwala, James P. Psaltis, Chinmay S. Marathe, Jessica A. Marathe, Peter J. Psaltis

**Affiliations:** 1 College of Medicine and Public Health, Flinders University, Adelaide 5042, Australia; richard.le@flinders.edu.au; 2 Heart and Vascular Program, Lifelong Health Theme, South Australian Health and Medical Research Institute, Adelaide 5000, Australia; tam.nguyen@sahmri.com (M.T.N.); momina.allahwala@sahmri.com (M.A.A.); jessica.marathe@sahmri.com (J.A.M.); 3 Department of Cardiology, Central Adelaide Local Health Network, Adelaide 5000, Australia; 4 Adelaide Medical School, Faculty of Health and Medical Sciences, The University of Adelaide, Adelaide 5005, Australia; james.psaltis@student.adelaide.edu.au (J.P.P.); chinmay.marathe@adelaide.edu.au (C.S.M.); 5 Department of Endocrinology, Central Adelaide Local Health Network, Adelaide 5000, Australia

**Keywords:** atherosclerosis, coronary artery disease, diabetes, glucagon-like peptide-1 receptor agonist, incretin, obesity

## Abstract

Owing to their potent glucose-lowering efficacy and substantial weight loss effects, glucagon-like peptide-1 receptor agonists (GLP-1 RAs) are now considered part of the frontline therapeutic options to treat both type 2 diabetes mellitus and nondiabetic overweight/obesity. Stemming from successful demonstration of their cardiometabolic modulation and reduction of major adverse cardiovascular events in clinical outcome trials, GLP-1 RAs have since been validated as agents with compelling cardiovascular protective properties. Studies spanning from the bench to preclinical and large-scale randomised controlled trials have consistently corroborated the cardiovascular benefits of this pharmacological class. Most notably, there is converging evidence that they exert favourable effects on atherosclerotic ischaemic endpoints, with preclinical data indicating that they may do so by directly modifying the burden and composition of atherosclerotic plaques. This narrative review examines the underlying pharmacology and clinical evidence behind the cardiovascular benefits of GLP-1 RAs, with particular focus on atherosclerotic cardiovascular disease. It also delves into the mechanisms that underpin their putative plaque-modifying actions, addresses existing knowledge gaps and therapeutic challenges and looks to future developments in the field, including the use of combination incretin agents for diabetes and weight loss management.

## 1. Introduction

As the leading cause of morbidity and mortality, cardiovascular disease (CVD) represents a formidable global disease burden [1]. In 2019, it was estimated that of 523 million people affected by all-cause CVD worldwide, there were 197 million prevalent cases of coronary artery disease (CAD), resulting in 9 million deaths [2]. Coronary artery disease, alongside peripheral artery disease (PAD) and ischaemic strokes, is a manifestation of atherosclerosis, a process whereby arteries undergo gradual stiffening and stenosis due to the build-up of inflammatory, fibro-lipidic plaques in their subintimal compartment [3].

As frequently recognised co-morbidities with atherosclerotic cardiovascular disease (ASCVD), type 2 diabetes mellitus (T2DM) and overweight/obesity are rapidly becoming pandemics of dysregulated metabolic health. T2DM currently affects 463 million people globally, a number predicted to increase by 25% in 2030 and 51% in 2045 [4]. Similarly, it has been projected that by 2030, 1.025 billion people will qualify as obese (BMI ≥ 30 kg/m^2^) [5]. This trajectory portends a myriad of future healthcare challenges. In particular, the burden of CVD will intensify, given that people with diabetes experience a two- to fourfold increased risk of developing ASCVD compared to those without [6] and that CVD accounts for two-thirds of deaths in the overweight/obese population [7]. The inextricable link between these disease states dictates that they cannot be managed in isolation.

Following the Food and Drug Administration’s (FDA) 2008 mandate that new diabetic drugs require a cardiovascular safety assessment, cardiovascular outcome trials (CVOTs) have been conducted to assess all new glucose-lowering pharmacotherapies [8]. In the case of glucagon-like peptide-1 receptor agonists (GLP-1 RAs), they have demonstrated significant reductions in major adverse cardiovascular event (MACE) endpoints, with the observed effects especially strong for reducing ischaemic events related to ASCVD [9]. The cardiovascular benefits of GLP-1 RAs are further supported by considerable preclinical and clinical research, which show that these agents exert favourable modulatory effects on atherosclerosis itself and its risk factors [10]. This narrative review examines the pharmacological properties and cardiovascular benefits of GLP-1 RAs, especially as they pertain to ASCVD. It also explores the spectrum of underlying mechanistic data for their anti-atherosclerotic properties, including those related to, but also independent of, cardiometabolic risk factors.

## 2. Pharmacology

### 2.1. GLP-1 Physiology 

In 1964, it was found that orally administered glucose elicits a significantly greater insulin response than parenterally administered glucose [11,12]. This phenomenon of exaggerated or amplified insulin response to oral glucose is now known as the ‘incretin effect’ and is substantially reduced in T2DM [13]. The incretin effect was attributed to incretin hormones, particularly glucagon-dependent insulinotropic polypeptide (GIP) and glucagon-like peptide-1 (GLP-1) [14,15]. GIP and GLP-1 are secreted from special entero-endocrine ‘K’ and ‘L’ cells, respectively, which are located predominantly in the small intestine and the colon [16].

After being post-translationally processed from proglucagon, GLP-1 is secreted and further converted in humans into its active forms, GLP-1(7–36) amide and GLP-1(7–37) [17]. In health, GLP-1 plasma levels increase fourfold following meals [17]. Postprandial secretion of GLP-1 follows a biphasic pattern: an early phase thought to be neurally mediated and a delayed phase after eating that is in direct response to luminal nutrients, particularly carbohydrates and fats [16,17]. The net result is insulin secretion and inhibition of glucagon secretion from islet β and α cells, respectively [14]. GLP-1 further limits postprandial glucose excursions by decelerating vagally mediated gastric emptying, which slows intestinal glucose absorption [17,18]. These canonical actions contribute to postprandial glucose control.

GLP-1 has been the incretin prioritised in drug discovery programs for T2DM. While GIP loses its insulinotropic action in experimental studies, even at supraphysiological doses, GLP-1 retains this effect under hyperglycaemic conditions [16]. Additionally, GLP-1 has indirect glucagonostatic effects that may assist in counteracting hyperglucagonaemia in T2DM [16,17], which may be mediated through upregulation of somatostatin secretion via somatostatin-2-receptor (SSTR2) [17]. As the insulinotropic effects are glucose-dependent and the counterregulatory response of glucagon to hypoglycaemia remains intact with GLP-1 infusion [19], the risk of hypoglycaemia is low. Hence, incretin-based drug development to control diabetic hyperglycaemia has been biased toward targeting the GLP-1 receptor (GLP-1R). 

### 2.2. Pharmacodynamics of GLP-1 Receptor Agonists 

#### 2.2.1. Insulin Secretion

**Figure 1:**
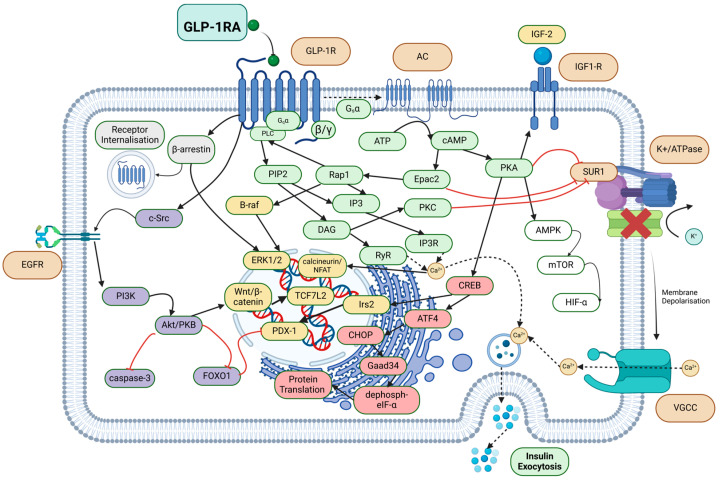
Intracellular signalling pathways induced by GLP-1 RA binding to GLP-1R in pancreatic β cells. These result in attenuation of endoplasmic reticulum (ER) stress (pink), inhibition of apoptosis (purple), increased insulin exocytosis (green), β cell proliferation (yellow) and improved glucose handling and homeostasis (white). AC: adenylyl cyclase, AKT/PKB: protein kinase B, AMPK: adenosine monophosphate-activated protein kinase, ATF4: activating transcription factor 4, ATP: adenosine triphosphate, B-raf: serine/threonine–protein kinase B-raf, Ca^2+^: calcium ions, cAMP: cyclic adenosine monophosphate, CREB: cAMP-response element binding protein, c-Src: tyrosine–protein kinase Src, DAG: diacylglycerol, dephosph-eIF2α: dephosphorylated eukaryotic initiation factor 2α, EGFR: epidermal growth factor receptor, Epac2: exchange protein activated by cAMP 2, ERK1/2: extracellular signal-regulated kinase 1/2, FOXO1: forkhead box protein O1, GLP-1R: glucagon-like peptide 1 receptor, GLP-1 RA: glucagon-like peptide 1 receptor agonist, HIF-α: hypoxia-inducible factor 1-α, IGF1R: insulin-like growth factor 1 receptor, IGF2: insulin-like growth factor 2, IP3: inositol 1,4,5-trisphosphate, IP3R: inositol 1,4,5-trisphosphate receptor, Irs2: insulin receptor substrate 2, mTOR: mammalian target of rapamycin, PDX-1: pancreatic and duodenal homeobox 1, PI3K: phosphoinositide 3-kinase, PIP2: phosphatidylinositol (4,5)-diphosphate, PLC: phospholipase C, PKA: protein kinase A, Rap1: Ras-proximate-1, RyR: ryanodine receptor, SUR1: sulfonylurea receptor 1, TCF7L2: transcription factor 7 like 2, VGCC: voltage-gated calcium channel, Wnt: wingless-related integration site. Created with BioRender.com.

Activation of the GLP-1R results in multiple signalling pathways that increase intracellular calcium, culminating in pancreatic insulin secretion (Figure 1). By imitating the endogenous action of GLP-1, GLP-1 RAs agonise the GLP-1R, a G protein-coupled receptor (GPCR). Once GLP-1 RAs bind, GTP-bound G_αs_ subunits from the GPCR complexes activate adenylyl cyclase, which converts ATP into cyclic adenosine monophosphate (cAMP). This activates protein kinase A (PKA), which phosphorylates the sulfonylurea receptor-1 (SUR1) subunit of the K^+^/ATPase channel, leading to early closure of this channel and an amplified physiological Ca^2+^ influx via voltage-gated calcium channels. Increased insulin secretion can also occur via G_q_/phospholipase C (PLC) signalling, where inositol trisphosphate 3 (IP_3_) binds to its receptor (IP_3_R) and diacylglycerol (DAG) binds to the ryanodine receptor to stimulate calcium-induced calcium release [17]. In a separate pathway, exchange protein directly activated by cAMP (Epac2) activates ras-proximate-1 (Rap1) [20], which activates PLC and increases downstream IP3/DAG levels, likewise inducing calcium release. Epac2 further enhances insulin exocytosis, as Epac2/Rim2/Piccolo complex binding to the Rab3–insulin interface enables exocytosis of insulin granules [17]. The calcium-permeable transient receptor potential melastatin 2 (TRPM2) channel has also been implicated in GLP-1-related insulin secretion via cAMP and PKA signalling [21]. Desensitisation of the GLP-1R is prevented through regular internalisation for intracellular trafficking and recycling, mediated by G_aq_ and β-arrestin pathways [22]. 

In addition to inducing insulin release, GLP-1R agonism can improve function and prevent exhaustion of pancreatic β cells on a transcriptional level by stimulating multiple pro-proliferative and anti-apoptotic pathways, while optimising intracellular metabolism (Figure 1). GLP-1 is thought to enable β islet mitogenesis via cyclin D2/Skp2 upregulation and p21/p27 downregulation, effectively reversing cell cycle arrest of pancreatic cells in hyperglycaemic states [23]. Similarly, GLP-1R agonism induces β cell proliferation and attenuates glucolipotoxicity through AMP-activated protein kinase (AMPK) and mammalian target of rapamycin (mTOR) signalling [24]. Upregulation of β cell transcription is likewise mediated via a variety of mechanisms including: increased levels of pancreatic and duodenal homeobox 1 (PDX-1) [25]; Wnt/β-catenin-mediated transcription factor 7-like 2 (TCF7L2) expression [17]; calcineurin/nuclear factor of activated T cell (NFAT) signalling [26]; increased insulin-like growth factor-1 receptor (IGF-1R) expression and membrane localisation [27]; increases in ERK1/2 due to GLP-1R-coupled β-arrestin recruitment and activation of Rap1 [17,28] and serine/threonine protein kinase B-raf [29]. Transactivation of epidermal growth factor receptor (EGFR) by GLP-1R occurs via c-Src, which induces phosphoinositide 3-kinase (PI3K) signalling [28] and prevents premature pancreatic cell apoptosis via upregulation of Bcl2 and inhibition of caspase-3 and forkhead box protein 1 (FoxO1) [28], thereby leading to disinhibition of PDX-1 [28]. GLP-1R activation further reduces endoplasmic reticular (ER) stress in β cells, preserving cellular function despite the cellular stresses induced by increased insulin biosynthesis [30] (Figure 1). PKA also activates cAMP response element-binding protein (CREB), which can, in turn, drive β cell growth by stimulating PDX-1 via insulin receptor substrate 2 (Irs2) signalling [17,31]. While CREB regulates the short-term pancreatic effects of GLP-1 RAs, mTOR and hypoxia-inducible factor-1α (HIF-1α) signalling mediate the secondary metabolic reprogramming that alters long-term glucose processing [32]. Collectively, the culmination of these activated pathways allows for enhanced pancreatic β cell viability and function. 

#### 2.2.2. Weight Loss and Appetite Suppression

Weight loss is a well-recognised and often beneficial effect of GLP-1 RA therapy. This has been attributed to the modulatory action of central appetite control, which results in caloric restriction and subsequent weight loss [33]. Within the hypothalamus, GLP-1-mediated activation of preproglucagon (Gcg^+^) neurons in the arcuate nucleus helps regulate appetite, independently of glucose control [34]. Animal studies have shown that GLP-1 can directly activate neurons containing pro-opiomelanocortin/cocaine- and amphetamine-stimulated transcription (POMC^+^/CART^+^), resulting in satiety [35]. GLP-1 can concurrently stimulate local inhibitory GABA neurons, which indirectly suppress neurons via agouti-related peptide/neuropeptide Y (AgRP^+^/NPY^+^) signalling, thereby reducing feelings of hunger. Control of satiety also occurs when GLP-1R^+^ neurons in the nucleus tractus solitarius of the hindbrain are stimulated, and this occurs independently of aversion caused by activation of GLP-1R^+^ neurons in the area postrema [36]. It is thought that vagal innervation is involved in the GLP-1 signalling behind appetite control, given that GLP-1 infusions increase vagal afferent firing and stimulate GLP-1R^+^ neurons in the nodose ganglion [37]. Furthermore, this theory of vagal dependency is bolstered by the nullification of GLP-1’s anorexic, gastrostatic and glucagonostatic effects in vagotomised patients [37]. There is also evidence that GLP-1 RAs can attenuate hyperstimulation of neural appetite and reward centres (insula, amygdala, putamen and orbitofrontal cortex) through direct GLP-1R agonism [38]. As such, the weight reduction that typically accompanies GLP-1 RA therapy is primarily due to appetite suppression, rather than increased energy expenditure or adipose tissue reduction. 

#### 2.2.3. Other Extra-Pancreatic Sites of GLP-1R Expression

**Figure 2:**
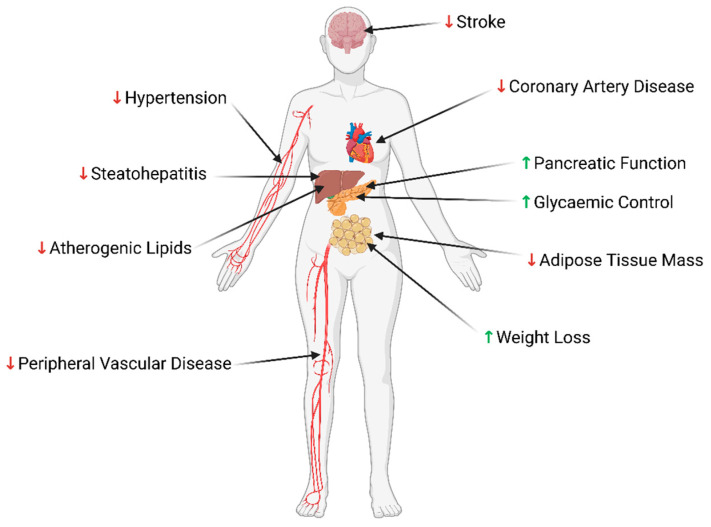
Summary of the cardiometabolic benefits of GLP-1 RA therapy. Green upward arrow: increases; Red downward arrow, decreases. Created with BioRender.com.

The vast range of extra-pancreatic effects exerted by GLP-1 RAs (Figure 2) may be due to the combination of multiple activated intracellular signalling pathways and the breadth of GLP-1R expression across the human body. Although predominantly found in the gastrointestinal and neural tracts, GLP-1R is also present in other tissues, including kidney, skeletal and smooth muscle, liver and adipose [39]. Within the cardiovascular system, it is expressed on cardiomyocytes and vascular smooth muscle and endothelial cells [39], along with circulating inflammatory cells [40]. This may contribute to the cardiovascular benefits achieved clinically with both synthetic GLP-1 and GLP-1 RA administration [41].

### 2.3. Pharmacokinetics of GLP-1 Receptor Agonists 

Native GLP-1 has limited clinical applications due to its labile nature, with rapid degradation by the dipeptidyl peptidase-4 (DPP-4) enzyme contributing to a short plasma half-life of two minutes [17]. GLP-1 RAs were thus designed as synthetic GLP-1 mimetics resistant to DDP-4 degradation, allowing a prolonged insulinotropic response for people with T2DM and thus limiting postprandial glucose excursions. This resistance to proteolytic degradation has been primarily achieved by alterations to the cleavage site of GLP-1, via substitution of alanine with alternative amino acids (Table 1). The endogenous half-life has also been increased through conjugation of the GLP-1 amide backbone with fatty acids, albumin, or human immunoglobulin to reduce the rate of renal elimination [42,43,44]. Therapeutic GLP-1 RAs developed to date have been predominantly administered via subcutaneous injection due to poor oral bioavailability, but the addition of sodium N-[8-(2-hydroxybenzoyl) amino caprylate (SNAC) as an absorption enhancer has circumvented this obstacle and allowed semaglutide to be given as an oral formulation [45] (Table 1).

One method of categorising drugs within the GLP-1 RA class has been according to their plasma half-life. With half-lives of 2–4 h, exenatide and lixisenatide are classed as short-acting agents (Table 1). As a result of their postprandial insulinotropic effects, these agents more closely resemble the effects of endogenous GLP-1. Long-acting GLP-1 RAs, such as liraglutide, dulaglutide and semaglutide in both oral and injectable forms, have half-lives longer than 12 h and primarily lower fasting blood glucose levels. 

Due to their inhibition of gastric emptying, GLP-1 RAs may delay the absorption of orally administered medications. However, besides prolonging the T_max_ value of other medications, they do not usually affect the pharmacokinetic parameters of other drugs, such as area under curve (AUC) or maximum concentration (C_max_) [46]. Although GLP-1 RAs do not typically cause hypoglycaemic episodes, there is an increased risk of developing hypoglycaemia when used in conjunction with insulin or sulfonylureas, meaning that dose adjustments may be needed [46].

The key adverse effects associated with GLP-1 RAs are gastrointestinal, such as nausea, vomiting, abdominal pain and diarrhoea [47]. These can represent obstacles to patient adherence, with up to 45% of GLP-1 RA users discontinuing therapy within five years [48]. To minimise adverse effects and improve adherence, GLP-1 RAs should be initiated at the lowest dose before gradually being uptitrated [10]. This improves tolerability, as gastrointestinal effects are most severe at initiation and generally wane over time. GLP-1 RAs are generally not recommended in patients with any history of gastrointestinal motility disorders (e.g., gastroparesis), pancreatitis, or pancreatic cancer [49]. There is also caution around using them in people with personal or family history of medullary thyroid carcinoma or multiple endocrine neoplasia type 2 (MEN2) syndrome. This stems from observations of thyroid C-cell hyperplasia in preclinical rodent studies; however, the same effect has not been replicated in large-scale human or primate studies [49]. Among other adverse effects, the SUSTAIN-6 trial of semaglutide also identified a signal for increased risk of diabetic retinopathy complications, but it remains unclear whether this is a class- or agent-specific effect or rather due to rapid HbA1c lowering [50]. The ongoing FOCUS trial (NCT03811561) will investigate this further by monitoring the progression of diabetic retinopathy in patients on semaglutide over a 5-year follow-up period. 

## 3. Cardiovascular Benefits of GLP-1 RAs

As a class, GLP-1 RAs are extremely effective at treating hyperglycaemia and lowering HbA1c levels in people with T2DM compared to other, well-established therapies, with HbA1c level reductions in the range of 0.5–1.5% compared to placebo [51]. In general, long-acting GLP-1 RAs have been demonstrated to be superior to short-acting GLP-1 RAs for glycaemic control, weight loss and tolerability [52]. Furthermore, head-to-head comparisons have shown that semaglutide and liraglutide are the most effective GLP-1 RAs for lowering HbA1c [51].

### 3.1. Cardiovascular Outcome Trials Data

#### 3.1.1. Composite Adverse Cardiovascular Endpoints

Cardiovascular outcome trials, which have been FDA-mandated since 2008 to identify potential adverse cardiovascular events caused by new anti-diabetic agents [50,53,54,55,56,57,58,59], have highlighted beneficial cardiovascular properties of GLP-1 RAs, albeit with some heterogeneity between different agents (Table 2). All GLP-1 RAs tested have demonstrated noninferiority for cardiovascular endpoints compared to placebo, thus underscoring their cardiovascular safety. Moreover, several have also achieved significant reductions compared to placebo in composite MACEs, usually comprising the endpoints of cardiovascular death, nonfatal myocardial infarction (MI) and nonfatal ischaemic stroke [50,54,56,57,59] (Table 2). Generally, long-acting agents have been associated with greater cardiovascular benefits than short-acting ones (Table 2).

Among the individual trials, the strongest benefit signals were seen with efpeglenatide and subcutaneous semaglutide, which lowered rates of MACEs by 27% (HR 0.73, 95% CI: [0.58, 0.92]) [59] and 26% (HR 0.74, 95% CI: [0.58, 0.95]) [50], respectively (Table 2). Meanwhile, albiglutide, dulaglutide and liraglutide were associated with statistically significant but more modest MACE reductions [54,56,57]. In contrast, the studies for exenatide, lisixenatide and oral semaglutide did not reach statistical significance for superiority compared to placebo [53,55,58]. However, oral semaglutide still reduced cardiovascular and all-cause death on secondary endpoint analysis [58]. The extent of oral semaglutide’s cardiovascular benefit is currently being further investigated with the SOUL trial (NCT03914326) (Table 3). 

Further to the individual study results, pooled meta-analysis of over 60,000 patients enrolled in the eight major phase 3 GLP-1 RA trials, of whom 72.4% had established CVD, concluded that GLP-1 RA therapy achieves a relative reduction in MACEs of 14% (HR 0.86, 95% CI [0.80–0.93], *p* < 0.0001) and all-cause mortality of 12% (HR 0.88, 95% CI [0.82–0.94], *p* = 0.0001) over a weighted average median follow-up of three years [9]. Secondary analyses of individual MACE components showed that GLP-1 RAs reduced cardiovascular death by 13% (HR 0.87, 95% CI [0.80–0.94], *p* = 0.001), total MI by 10% (HR 0.90, 95% CI [0.83–0.98], *p* = 0.020) and total strokes by 17% (HR 0.83, 95% CI [0.76–0.92], *p* = 0.0002) [9]. A separate meta-analysis has found this MACE benefit is biased towards patients with preestablished cardiovascular disease, with reductions in MACEs of 15% in people with ASCVD (OR 0.85, 95% CI [0.81–0.90], *p* = 0.00001) compared to 6% in people with multiple cardiovascular risk factors but no ASCVD (OR 0.94, 95% CI [0.83–1.06], *p* = 0.31) [60]. 

These benefits position GLP-1 RAs as a suitable addition to the repository of secondary prevention therapies for cardiovascular disease. Another meta-analysis comparing nine diabetic drug classes recently found that in patients with T2DM at increased cardiovascular risk on metformin-based background treatment, GLP-1 RAs and SGLT2 inhibitors stood apart as reducing all-cause mortality and cardiovascular death [61]. Notably, whereas SGLT2 inhibitors reduced heart failure hospitalisation, semaglutide and dulaglutide were associated with lower risk of stroke [61]. It is also important to highlight that the evidence base for GLP-1 RAs in the different trials was achieved on other background cardioprotective therapies. For example, in SUSTAIN-6 64% of participants were also taking aspirin, 21% clopidogrel or ticagrelor, 77% lipid-lowering medication (mostly statins) and 94% anti-hypertensives with 50% on ACE inhibitors [50]. The magnitude of event reduction with GLP-1 RAs, especially semaglutide, also compares favourably with the effectiveness of other drugs commonly used to mitigate cardiovascular risk. By way of reference, a 2011 systematic review of aspirin’s use for primary prevention of MACEs in patients with diabetes demonstrated an overall risk ratio of 0.91 [62]. Meanwhile, statins are recognised to reduce major vascular events by ~21% for every 1.0 mmol/L reduction in LDL-C [63], which is similar to the overall benefit from the ACE inhibitor, ramipril, in the HOPE study where almost 40% of participants had diabetes [64]. Importantly, the effectiveness of GLP-1 RAs for reducing cardiovascular events also sits comfortably alongside other nonglucose-targeting therapies that have been introduced more recently for ASCVD. These include PCSK9 inhibitors (e.g., evolocumab [65]) and anti-inflammatory agents, such as colchicine [66].

When interpreting the results of the GLP-1 RA outcome trials, it is important to note that there was heterogeneity between studies, including differences in baseline cardiovascular risk between trial cohorts. Recruitment in most of the trials was enriched for patients with established CVD to ensure adequate statistical power within short follow-up timeframes, such as in AMPLITUDE-O (efpeglenatide) and SUSTAIN-6 (subcutaneous semaglutide) where >80% had preexisting CVD [50,59]. In contrast, REWIND (dulaglutide) recruited participants with comparatively lower cardiovascular risk, as it had the lowest baseline mean HbA1c of 7.2% and only 31.5% had known CVD [57]. Another characteristic of study design that might confound comparisons between different agents is the temporal difference in excluding patients in the early post-MI period. For instance, LEADER (liraglutide) excluded patients who had suffered a stroke or acute coronary syndrome (ACS) in the two weeks before recruitment [54], whereas SUSTAIN-6 set this exclusion window to 90 days prior [50]. As patients are at highest risk of recurrent ischaemic events in the early post-MI period [67], such differences in exclusion criteria can influence the cardiovascular event rates captured by different studies. For example, the nonbenefit for lixisenatide in the ELIXA trial may be partially attributable to its extremely high-risk cohort, where 100% of participants had established CVD and an ACS within the last 180 days [53]. Hence, it is difficult to draw firm conclusions about the relative merits of different GLP-1 RAs for cardiovascular protection.

#### 3.1.2. Specific Endpoints of Interest

##### Ischaemic Heart Disease

With respect to individual cardiovascular endpoints, most of the trials have shown benefits in CAD-related outcomes. As described above, meta-analysis of outcome trials has demonstrated a mean relative reduction of 10% in the rates of new and recurrent MI by agents across the GLP-1 RA class [9]. Discounting the trials that did not show benefit for MI, this reduction ranged from 12% for liraglutide in the LEADER trial [54] up to 25% for albiglutide in the HARMONY OUTCOMES trial [56]. Moreover, in the AMPLITUDE-O, HARMONY OUTCOMES and SUSTAIN-6 trials [50,56,59], there were significant reductions in the expanded composite outcome, defined as hospitalisations for unstable angina and urgent coronary revascularisations. Notably, in SUSTAIN-6, subcutaneous semaglutide was associated with 35% reduction in revascularisation procedures of either the coronary or peripheral vessels (HR 0.65, 95% CI: [0.50, 0.86]) [50].

##### Stroke 

GLP-1 RAs have also been linked to significant reductions in cerebrovascular events. By comparison to placebo, dulaglutide (REWIND) and subcutaneous semaglutide (SUSTAIN-6) resulted in relative reductions in nonfatal stroke of 24% and 39%, respectively [50,57]. However, this was not observed in other GLP-1 RA trials. Importantly, meta-analysis has indicated that the salutary effects on stroke outcomes were driven by reductions in ischaemic rather than haemorrhagic stroke [68]. In the primary prevention setting, GLP-1 RAs have been found to reduce the incidence of both nonfatal and total strokes by around 16% [69]. Indeed, GLP-1 RAs and thiazolidinediones are the only two classes of diabetic drugs that have been shown to reduce stroke risk [70].

##### Heart Failure 

As a class, GLP-1 RAs do not reduce heart failure hospitalisations to the same extent as SGLT2 inhibitors, which can reduce heart-failure-related hospitalisations by ~30% [71]. Among the GLP-1 RA outcome trials, only AMPLITUDE-O showed significant reduction in risk of heart-failure-related hospitalisations with a hazard ratio of 0.61 (95% CI [0.38, 0.98]) [59]. However, the recent STEP-HFpEF study found that subcutaneous semaglutide is associated with both symptomatic and functional improvement in patients with nondiabetic obesity (body mass index, BMI ≥ 30 kg/m^2^) and heart failure with preserved ejection fraction (HFpEF) [72]. After 52 weeks of semaglutide at up to 2.4 mg once weekly, there was significant weight loss, with an estimated different of 10.7 percentage points compared to placebo (*p* < 0.001). Semaglutide was also associated with improvements in a hierarchical composite endpoint, comprising death, heart failure events, differences in the Kansas City Cardiomyopathy Questionnaire clinical summary score (KCCQ-CSS) and 6 min walk distance (win ratio, 1.72; 95% CI [1.37–2.15]; *p* < 0.001) [72]. Furthermore, there were also reductions in systolic blood pressure (BP), C-reactive protein (CRP) and N-terminal pro-B-type natriuretic peptide (NT-proBNP) [72]. Whether semaglutide’s effects on these parameters simply reflect the benefits of weight loss, as opposed to *bona fide* improvement in HFpEF, remains unclear and requires further evaluation. 

##### Peripheral Artery Disease 

Given that atherosclerosis also manifests in noncoronary territories, the effect of GLP-1 RAs on the peripheral arterial vasculature is also of major importance. However, in contrast to CAD and stroke, there are much fewer specific data on the effect of GLP-1 RAs on PAD-related outcomes. Each of the major outcome trials (SUSTAIN-6, EXCEL, HARMONY OUTCOMES, REWIND) was underpowered for these events, as the proportion of patients with CAD consistently outnumbered those with PAD [50,55,56,57]. For example, PAD was underrepresented in both LEADER and SUSTAIN-6, affecting only 12.7% and 14.0% of participants at baseline, respectively [73]. Moreover, ELIXA did not feature PAD at all in its inclusion criteria [53]. Furthermore, revascularisation outcome data were also reported as an aggregate of coronary and peripheral interventions, preventing targeted analysis of PAD-related outcomes. As such, future studies with PAD-enriched cohorts and prespecified PAD outcome analyses are required. 

Nevertheless, the currently available data do suggest that GLP-1 RAs are safe and associated with a reduction in PAD events [74]. An observational real-world study of two geographically separated Italian cohorts found that GLP-1 RA use decreased rates of PAD (Lombardy cohort: HR 0.72, 95% CI [0.64–0.82]; Apulia cohort: HR 0.80, 95% CI 0.67–0.98) and lower limb complications (Lombardy cohort: HR 0.67, 95% CI [0.56–0.81]; Apulia cohort: HR 0.69, 95% CI [0.51–0.93]) [75]. This was supported by a Danish cohort study of 309,166 patients with diabetes, which similarly showed that GLP-1 RAs were associated with a 50% lower rate of diabetes-associated amputations compared to those not on GLP-1 RA treatment (HR 0.50, 95% CI [0.54–0.74], *p* < 0.005) [76]. With these signals of safety and possible benefit for PAD-related outcomes, GLP-1 RAs could potentially be adopted as the preferred therapy over other anti-diabetic medications in patients with concurrent T2DM and PAD [77]. This is especially the case given that the CANVAS program identified a twofold increased risk of lower limb amputation with the SGLT2 inhibitor canagliflozin compared to placebo [78]. Indeed, a recent meta-analysis suggested that GLP-1 RAs were associated with significantly lower incidence of lower limb amputations than SGLT2 inhibitors (3.54 ± 3.18 versus 4.72 ± 3.99 events per 1000 patient-years, *p* = 0.004) [79]. Moving forward, several studies (e.g., STRIDE, STARDUST, LEADPACE) are set to expand upon this area of knowledge by investigating how GLP-1 RAs may affect functional movement and peripheral endothelial function in patients with PAD (Table 3). 

### 3.2. Observational Cardiovascular Data and Real-World Experience

Real-world experience has also been reassuring and congruent with CVOT findings about the safety and benefits of GLP-1 RAs in wider populations of high-cardiovascular-risk patients. Svanström et al. performed a retrospective registry-based cohort analysis of 46,804 patients with T2DM in Denmark and Sweden, who were evenly distributed between the use of liraglutide and DDP-4 inhibitors [80]. Compared to those on DDP-4 inhibitors, patients using liraglutide had a 10% lower risk of MACEs, 22% lower risk of cardiovascular death (unadjusted HR 0.78, 95% CI: [0.68, 0.91]) and 17% lower risk of all-cause death (unadjusted HR 0.83, 95% CI: [0.77, 0.90]) [80]. These results also held with a sensitivity analysis that accounted for HbA1c, smoking status, BP, albuminuria, estimated glomerular filtration rate (eGFR) and BMI [80]. Meanwhile, Trevisan et al. studied 17,868 patients with T2DM and prior MI from the SWEDEHEART registry. Although only used in 2% of the cohort, GLP-1 RAs were associated with 28% lower event risk over a median follow-up of 3 years compared to standard diabetic care (95% CI [0.56, 0.92]) [81]. This was mostly driven by reductions in stroke and re-infarction and was consistent after propensity score matching and across different subgroups. Given the high-fidelity environment of these real-world studies, the results are generalisable to wider populations.

Given the findings of the CVOTs and recent real-world experience, the use of GLP-1 RAs has now become guideline-recommended by the European Society for Cardiology, American Diabetes Association and European Association for the Study of Diabetes in patients with T2DM and concurrent ASCVD or at high risk of ASCVD, such as those with target organ damage or multiple cardiovascular risk factors [82,83]. These agents can be commenced either firstline in drug-naïve patients or secondline in patients already using metformin or other glucose-lowering agents, including insulin. 

## 4. Does Risk Factor Modulation Explain the Cardiovascular Benefit of GLP-1 RAs? 

The reductions in cardiovascular outcomes associated with GLP-1 RAs appear to be driven predominantly by reductions in atherosclerotic and/or ischaemic events. However, it is unclear to what extent this is simply mediated by improvements in cardiovascular risk factors, including glycaemia and weight loss, or if additional mechanisms are involved. 

### 4.1. Glycaemic Control 

Diabetic hyperglycaemia fosters a pro-inflammatory and oxidative microenvironment that is conducive to the development of ASCVD and its ischaemic complications [84]. While better glycaemic control has long been associated with improved microvascular complications of T2DM, it has been more difficult to demonstrate this relationship for ASCVD (or macrovascular) outcomes [85]. Mediation analyses suggest that glucose lowering may only partially account for the cardiovascular benefits of GLP-1 RA therapy. For example, it has been determined from the LEADER and REWIND trials that HbA1c lowering contributed to 36–41% of cardiovascular event reduction [86,87]. It is also worth noting that in these analyses, changes in other metabolic variables, such as bodyweight, systolic BP and low-density lipoprotein cholesterol (LDL-C), did not reach significance for mediation [86,87]. Other analyses suggest that any MACE reductions associated with HbA1c lowering are predominantly driven by reductions in nonfatal stroke [88].

### 4.2. Weight Loss 

Weight loss can favourably modify cardiometabolic risk factors [89] and reverse the chronic inflammation of an obesogenic microenvironment [90]. Known to be a positive prognostic factor for cardiac outcomes, weight loss also lowers the incidence of clinical endpoints, such as urgent revascularisations, acute ASCVD events and total mortality [91]. Diabetic obesity has also been independently associated with coronary plaque burden, emphasising the need to target this cardiovascular risk factor [92].

Although GLP-1 RAs are one of the most effective agents within the existing pharmacological armamentarium for obesity [93], the degree of weight loss varies between different agents (Table 2). In SUSTAIN-6, 0.5 mg and 1.0 mg doses of subcutaneous semaglutide resulted in mean weight loss of 3.6 kg and 4.9 kg from baseline, compared to 0.7 kg and 0.5 kg with placebo, respectively [50]. For patients with overweight or obesity who did not have T2DM, 2.4 mg of semaglutide in the STEP1 trial led to mean bodyweight reduction of 14.9% (2.4% with placebo), with 86% and 51% of participants achieving ≥5% and ≥15% bodyweight reductions, respectively [94]. Real-world data have shown that high doses of semaglutide (1.7 mg and 2.4 mg) are associated with mean bodyweight percentage reductions of 6% after three months and 12% after six months [95].

It is therefore tempting to causally link the cardiovascular benefits of GLP-1 RA therapy with its associated weight loss. However, weight loss appears to be mostly independent of MACE-lowering effects, as seen in the outcome study data. This is highlighted by similar reductions for primary MACE composite outcomes with dulaglutide and albiglutide, which only yield modest weight loss, and semaglutide and liraglutide, which achieve more substantial weight loss (Table 2). Furthermore, the magnitude of GLP-1 RA-induced weight loss does not fully account for the degree of cardiovascular benefit observed. Bodyweight reductions of 2–5 kg have been shown previously in epidemiological studies to be insufficient to achieve the degree of cardiovascular risk reduction seen in the GLP-1 RA outcome trials. Notably, intensive lifestyle intervention for weight loss in overweight/obese patients did not provide mortality benefit in the LOOK AHEAD trial [96], although *post hoc* analysis did find that >10% weight loss within the first year of intervention was associated with a 21% reduction in cardiovascular mortality [97]. The Swedish Obese Subjects study supported this, finding that modest weight loss of 5 kg was not enough to alter CVD risk, whereas 10–44 kg weight loss in the setting of bariatric surgery was needed for significant risk factor reduction at 10-year follow-up [98]. A systematic review concurred with these epidemiological observations, concluding that bariatric surgery is superior to GLP-1 RA therapy with regard to weight loss (mean difference −22.68 kg) and BMI reduction (mean difference −8.18 kg/m^2^) [99]. Retrospective cohort studies have found that due to the magnitude of weight loss, bariatric surgery is superior to GLP-1 RAs for reducing MACEs, although the use of second-generation GLP-1 RAs such as semaglutide was underrepresented in these studies [100,101]. 

The impetus to prescribe GLP-1 RAs primarily for obesity is continually growing. Although the cardiovascular benefits of using GLP-1 RAs for diabetic patients have been well-documented [9], there was previously a paucity of evidence for using these agents for cardiovascular risk reduction in nondiabetic overweight/obesity. With 17,604 participants aged ≥ 45 years across over 800 sites, the SELECT trial recently showed that once-weekly subcutaneous semaglutide 2.4 mg significantly lowered MACEs by 20% compared to placebo for overweight and obese patients (BMI ≥ 27) with preexisting ASCVD but without diabetes [91] (Table 3). This is in addition to *post hoc* analysis showing that weight loss was sustained in this cohort for up to four years [102]. An early divergence of the time-to-first-event curves within three months of treatment initiation in SELECT means that the onset of semaglutide’s cardiovascular benefits preceded most of the achieved weight loss or other changes to cardiometabolic risk factors [91]. This is important because it suggests that mechanisms beyond just weight loss are helping to mediate the cardioprotective effects of GLP-1 RAs [91]. Consequently, although semaglutide 2.4 mg (Wegovy) had already been approved in 2021 for chronic weight management in adults with obesity or overweight and at least one weight-related condition, the FDA has now expanded this indication to cardiovascular risk reduction in people with concurrent CVD and overweight/obesity.

### 4.3. Blood Pressure 

All outcome trials also reported reductions in systolic BP [50,53,54,55,56,57,58,59], ranging from an average of 0.65 mmHg with albiglutide [56] to 2.57 mmHg with 1.0 mg subcutaneous semaglutide [50]. Meanwhile, changes in diastolic BP were variable and not significant. A meta-regression analysis has further clarified that reductions in systolic BP are independent of weight loss and HbA1c improvements, raising the point that GLP-1 RAs have an independent anti-hypertensive effect [103].

### 4.4. Lipids 

GLP-1 RAs generally have a positive yet modest effect on blood lipid profiles. In PIONEER 6, oral semaglutide was associated with a reduction of 4–5% in total cholesterol and LDL-C levels and 12% in triglyceride levels from baseline [58]. Similarly, subcutaneous semaglutide in SUSTAIN-6 reduced triglycerides by 7–8% and both total cholesterol and LDL-C by 3% compared to baseline levels [50]. Other trials reported statistically, but not clinically, significant improvements in LDL-C levels [55,57,59]. GLP-1 RAs have also shown potential for use as an adjunctive lipid control measure within real-world settings. For example, liraglutide combined with metformin lowered levels of atherogenic lipoproteins in patients with T2DM and CAD who were already taking statins [104]. Postprandial reductions in remnant cholesterol, an aggregate of very low-density lipoprotein (VLDL) and intermediate-density lipoprotein (IDL), may be mediated by liraglutide-driven reductions of ApoC III [105]. Moreover, detailed lipidomic analysis revealed that liraglutide significantly lowers blood concentrations of multiple lipid species, including ceramides, phosphatidylcholines, phosphatidylethanolamines and triglycerides [106]. Meta-analysis data have also associated GLP-1 RAs with modest lowering of total cholesterol, LDL-C and triglyceride levels, but direct causality between GLP-1 RA-mediated lipid-lowering effects and improved cardiovascular outcomes has not been adequately investigated [107].

The totality of clinical evidence so far suggests that the cardiovascular benefits of GLP-1 RAs cannot be easily attributed to changes in a single risk factor, whether it be glycaemia, weight or other cardiometabolic factors. This has led researchers to explore the effects of GLP-1 RAs on other mechanistic drivers of ASCVD (Table 2).

## 5. Evidence of GLP-1 RAs’ Direct Impact on Atherosclerotic Plaques 

Numerous preclinical models suggest that the effect of GLP-1 RAs on reducing ischaemic macrovascular events is mediated through direct attenuation of the underlying atherosclerotic substrate. Murine models have particularly contributed to this evidence. Liraglutide and semaglutide both resulted in decreased plaque area and aortic intimal thickening in atherosclerotic models in *Apoe*^−/−^ and *Ldlr*^−/−^ mice, with this occurring independently of weight loss and cholesterol-lowering effects [108]. Although both early and late initiation of dulaglutide reduced plaque area in *Apoe*^−/−^ mice, intervention at early- but not late-stage diabetes was associated with significantly reduced macrophage infiltration into aortic root lesions [109]. Hence, greater attenuation of plaque inflammation may be seen if GLP-1 RAs are administered earlier in the diabetic and atherosclerotic process [109].

In addition to murine research, a study in *Ldlr*^−/−^ Watanabe heritable hyperlipidaemic (WHHL) rabbits showed that lixisenatide promoted aortic plaque stability, with increased fibrotic tissue and reduced necrotic and calcified areas within plaques, relative to the control group [110]. However, despite these preclinical observations it is interesting that lixisenatide did not reduce MACEs in the ELIXA trial [53]. This discrepancy could potentially be explained by the use of osmotic mini-pumps in the rabbit study, which may have artificially improved the pharmacokinetic efficacy of lixisenatide by circumventing its short half-life [111].

Although there appears to be a positive association between serum GLP-1 levels in patients with CAD and their degree of coronary plaque burden, the causal nature and clinical significance of this finding remain unclear [112]. Several human studies are underway to investigate if GLP-1 RAs directly attenuate plaque formation or at least modulate the composition of plaques to a more passive or stable phenotype (Table 3). The Semaglutide Treatment on Coronary Plaque Progression (STOP) trial (NCT03985384) used serial coronary computed tomography angiography (CCTA) to explore the effect of subcutaneous semaglutide on coronary plaque progression in patients with T2DM who had established ASCVD or at least one additional CVD risk factor [113]. Although unpublished data indicated no significant reduction in plaque volume, there was a signal for a ‘stabilisation’ effect, whereby semaglutide was associated with increased conversion of noncalcified plaque to calcified plaque. The ongoing SAMAS trial (NCT05147896) will examine how oral semaglutide affects parameters of ASCVD, such as arterial stiffness, carotid intima–media thickness and endothelial function, along with atherosclerotic risk factors (e.g., LDL-C, HbA1c, high-sensitivity CRP) [114].

## 6. Putative Anti-Atherosclerotic Mechanisms 

**Figure 3:**
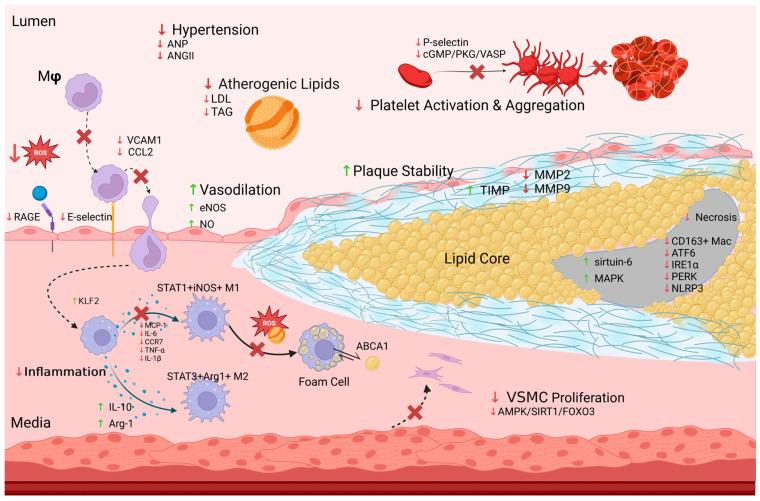
Putative Molecular Mechanisms for Anti-Atherogenic Effects of GLP-1 RAs. ABCA1: ATP-binding cassette transporter A1, ANG-II: angiotensin-II, AMPK: AMP-activated protein kinase, ANP: atrial natriuretic peptide, Arg-1: arginase-1, ATF6: cyclic AMP-dependent transcription factor-6, CCL2: C–C motif chemokine ligand 2, CCR7: C-C chemokine receptor type 7, CD62p: P-selectin, CD163+ Mac: cluster of differentiation 163+ macrophage, cGMP: cyclic guanine monophosphate, CRP: C-reactive protein, eNOS: endothelial nitric oxide synthase, FOXO3a: forkhead transcription factor O subfamily member 3a, HTN: hypertension, iNOS: inducible nitric oxide synthase, IFN-γ: interferon-γ, IL-1β: interleukin-1β, IL-6: interleukin-6, IL-10: interleukin-10, IRE1α: inositol-requiring enzyme 1, JNK: c-Jun N-terminal kinase, KLF2: Krüppel-like factor 2, LDL-C: low-density lipoprotein cholesterol, MAPK: mitogen-activated protein kinase, Mφ: macrophage, M1: M1 macrophage, M2: M2 macrophage, MMP: matrix metalloproteinase, MCP-1: monocyte chemoattractant protein-1, NLRP3: nucleotide-binding domain (NOD)-like receptor protein 3, NO: nitric oxide, PERK: protein kinase R-like ER kinase, PKG: protein kinase G, RAGE: receptor for advanced glycation end-products, ROS: reactive oxygen species, SIRT1: sirtuin 1, STAT1: signal transducer and activator of transcription 1, STAT3: signal transducer and activator of transcription 3, TAG: triacylglyceride, TIMP: tissue inhibitor of metalloproteinases, TNF-α: tumour necrosis factor-α, VASP: vasodilator-stimulated phosphoprotein, VCAM1: vascular cell adhesion protein 1, VSMC: vascular smooth muscle cell. Green upward arrow, increases. Red downward arrow, reduces. Red cross, inhibits. Created with BioRender.com.

Increasing evidence shows that GLP-1 RAs exert pleiotropic effects on different mediators of atherogenesis, which may help explain their benefits on ASCVD events beyond cardiometabolic risk factor control (Figure 3).

### 6.1. Correcting Vascular Dysfunction 

An intact and functioning endothelium is crucial for maintaining physiological vascular function, with endothelial dysfunction being the first step of the atherogenic cascade. GLP-1 is known to possess vasodilatory effects, due to its direct actions on vascular endothelium. Infusion of GLP-1 has been associated with favourable endothelial function and nitric oxide synthase (NOS)-mediated vasodilation [115]. There is also evidence that it causes vasodilation independent of adenosine [116]. Furthermore, GLP-1 RA-mediated vasodilation can occur in states of low Nox-1/endothelin-1 and normal eNOS/guanylyl cyclase expression [117]. In a mouse study, GLP-1 RA therapy was also found to delay the progression of atherosclerosis through AMP-activated protein kinase (AMPK)-dependent arrest of angiotensin-II-induced vascular smooth muscle cell (VSMC) proliferation [118]. Through targeting AMPK/SIRT1/FoxO3a pathways, healthy vascular function is achieved by maintaining a calponin^+^SM22α^+^ VSMC phenotype [119].

With particular focus on diabetic angiopathy, GLP-1 RAs may also attenuate atherosclerotic progression due to the vasoprotective effects of reducing advanced glycosylation end-products (AGEs). In addition to anti-glucolipotoxic effects on the endothelium [120], treatment in *Apoe*^−/−^ mice inhibits formation of the receptor for advanced glycosylation end-products (RAGE) in the aorta and lowers serum AGE levels [121]. This presents a new opportunity for the treatment of vascular complications associated with diabetes, since the AGE/RAGE interaction is a key component in the progression of diabetic atherosclerosis.

### 6.2. Targeting Diabetic Dyslipidaemia 

Moderating lipid levels is crucial to reducing atherogenesis [122]. GLP-1R agonism modulates different steps of cholesterol homeostasis on both genetic and protein levels. It results in downregulation of lipogenic genes and upregulation of lipolysis in human adipocytes, attenuating a systemic obesogenic state [123]. The GLP-1R is also involved in cholesterol efflux from foam cells mediated by the ATP-binding cassette transporter (ABCA1) [124]. GLP-1 RAs can further downregulate acetyl-CoA acetyltransferase 1 (ACAT1) expression [125] and upregulate signalling between adaptor protein phosphotyrosine interacting with pleckstrin homology (PH) domain and leucine zipper 1 (APPL1) and adiponectin, which suppresses foam cell formation [126]. Whilst the effects of GLP-1 RA administration alone on lipogenesis have not been studied, co-administration of GLP-1 RA with glucagon reduces lipogenesis by lowering expression of β-hydroxy β-methylglutaryl-CoA (HMG-CoA) reductase and sterol regulatory element-binding protein-1c (SBREBP-1C) [127]. Furthermore, GLP-1/glucagon co-agonism improves reverse cholesterol transport and bile acid homeostasis through increased expression of LDLR/ABCA1 and cytochrome P450 family 7 subfamily A member 1 (CYP7A1)/ATP-binding cassette, subfamily B member 11 (ABCB11), respectively [127]. Finally, GLP-1 RAs may have a role in treating nonalcoholic steatohepatitis through lowering hepatic inflammation, steatosis and fibrosis, although the mechanistic basis for this is yet to be determined [128].

### 6.3. Dampening Inflammation

A chronically dysregulated inflammatory response underpins the progression of atherosclerosis [129]. It is thought that GLP-1 RAs also mediate atheroprotective effects by dampening inflammation systemically, as well as locally in the arterial wall. To this end, they have been shown to downregulate transcriptional expression of genes involved in both inflammation and oxidative stress. In addition to reductions in plasma levels of interferon-γ (IFN-γ) and tumour necrosis factor-α (TNF-α), semaglutide also lowers mRNA levels of other cytokines, such as interleukin-6 (IL-6), chemokine ligand 2 (CCL2) and vascular cell adhesion molecule-1 (VCAM-1), which are involved in leukocyte recruitment and extravasation [108]. Furthermore, GLP-1R agonism can reduce gene transcription of nuclear factor kappa-B (NF-κB) and superoxide dismutase 2 (SOD2) by reversing the hyperglycaemia-induced DNA demethylation [130].

Liraglutide was found to confer resistance to TNF-α- and liposaccharide-induced inflammation and inhibit monocyte recruitment to the vascular endothelium by lowering VCAM-1 and E-selectin expression in cultured human aortic endothelial cells [131]. This was speculated to result from increased calcium/calmodulin-dependent protein kinase I (CaMKI)/CREB levels and induction of calcium/calmodulin-dependent protein kinase kinase-β (CaMKKβ)/AMPK signalling [131]. Furthermore, through the extracellular-signal-regulated kinase 5 (ERK5) pathway, GLP-1 RAs have been shown to increase Krüppel-like factor 2 (KLF2) levels and prevent inhibition of mitogen-activated protein kinase (MAPK), which causes downstream anti-inflammatory effects, including decreased leukocyte adhesion [132]. GLP-1 RAs can additionally guard against hyperglycaemia-induced autoinflammatory damage, inhibiting NLR family pyrin domain-containing 3 (NLRP3) inflammasome formation and thereby conferring anti-pyroptotic effects on cardiomyocytes [133]. 

There is also evidence for modulatory effects of GLP-1 RAs on macrophage biology. In addition to reducing plaque size and necrotic core area in *Apoe*^−/−^ *Irs2*^+/−^ mice, lixisenatide was found to result in a greater proportion of M2-like anti-inflammatory (STAT3^+^Arginase-1^+^) macrophages in aortic plaques, with fewer M1-like, pro-inflammatory (STAT1^+^iNOS^+^) macrophages [134]. Similarly, liraglutide increased M2-like macrophage populations in *Apoe*^−/−^ mice and increased aortic plaque expression of the anti-inflammatory mediators IL-10 and Arg-1 [135]. Notably, the oxidative-stress-sensitive channel TRPM2 has also recently been shown to play a role in atherogenesis by promoting macrophage recruitment, foam cell formation and vascular inflammation within atherosclerotic plaques [136,137]. Similarly, it can exacerbate N-methyl-D-aspartate (NMDA) receptor-related excitotoxicity and neuronal death in ischaemic stroke [138]. Given TRPM2’s role in both atherosclerosis and GLP-1-induced insulin secretion as discussed above [21], further research on GLP-1/TRPM2 interactions could enable further pharmacological targeting of macrophage-related inflammation in diabetes and ASCVD. 

Despite these potential anti-inflammatory mechanisms, molecular imaging studies have yielded discrepant results about whether GLP-1 RAs can attenuate vascular wall inflammation. Positron emission tomography (PET) imaging of rabbits identified that semaglutide reduced macrophage activation and metabolism in the aortic wall by using [^64^Cu]Cu-DOTATATE and [18F]FDG radiotracers, respectively [139]. Conversely, the LIRAFLAME trial failed to show a significant effect of liraglutide compared to placebo on vascular inflammation as assessed by [18F]FDG PET after 26 weeks of treatment in people with T2DM [140]. To clarify these discrepant results, further human imaging studies are needed to elucidate the effects of GLP-1 RAs on atherosclerotic plaque burden and composition. 

### 6.4. Reduction in Endoplasmic Reticulum Stress 

In addition to reducing endoplasmic reticular (ER) stress in pancreatic β cells in diabetes, GLP-1 RAs can also alleviate the lipotoxic stress that the atherosclerotic milieu chronically places on the ER in macrophages and endothelial cells, thereby helping to mitigate the formation of necrotic cores that are characteristic of unstable plaques [141]. This has been demonstrated through a reduction in levels of inositol-requiring enzyme 1α (IRE1α) and its downstream target c-Jun N-terminal kinase (JNK), which are markers of acute ER stress [142]. Liraglutide was also shown to reverse dextrose- and tunicamycin-induced ER stress in human coronary artery endothelial cells by downregulating the expression of glucose-regulated protein 78 (GRP78)/ATF6 and phosphorylation of protein kinase RNA-like endoplasmic reticulum kinase (PERK)/IRE1α, and this occurred to a greater extent than with SGLT2 inhibition or metformin [143]. In other work, the activation of p38 MAPK by exendin-4 was proposed to protect against lipoapoptosis [144]. Finally, there is also evidence to support that GLP-1R agonism can stabilise plaques not only by limiting inflammation and oxidative stress but also by increasing the expression of sirtuin 6, a DNA repair deacetylase [145]. Taken together, the ability of GLP-1 RAs to limit cellular damage mediated by ER stress is another mechanism by which they may help attenuate atherogenesis and passivate plaques to prevent plaque rupture and atherothrombotic events. 

### 6.5. Regulating Adiponectin and Other Adipokines 

Adipokine dysregulation, which presents as hypoadiponectinaemia and hyperleptinaemia, contributes to the insulin resistance seen in T2DM. In fact, low serum adiponectin levels may be an independent predictor for future ASCVD events and a marker of CAD severity [146]. To this end, GLP-1 RAs, such as exendin-4, have been shown to increase adiponectin levels via Sirt1/FoxO1 signalling [147]. Moreover, a meta-analysis of 20 randomised controlled trials revealed that, independent of fat mass changes, liraglutide significantly increased circulating adiponectin levels [148].

### 6.6. Modification of Epicardial Adipose Tissue 

Epicardial adipose tissue (EAT), which is a store of visceral fat located between the myocardium and pericardium, has been implicated in the pathogenesis of ASCVD and has been shown to confer a pro-inflammatory milieu for the neighbouring coronary arteries [149]. With GLP-1R expressed in EAT at much higher levels than in subcutaneous tissue [150], GLP-1 RAs may be able to target EAT for cardiovascular benefit. Dose-dependent reductions in EAT thickness have been observed for both semaglutide and dulaglutide in obese patients with T2DM [151]. This was confirmed via pooled analysis, with a mean reduction of 1.83 mm in EAT thickness following GLP-1 RA use [152]. This presents an exciting and novel therapeutic target for ASCVD prevention and treatment, as reductions in pericoronary EAT have been shown in mechanistic studies to attenuate coronary atherosclerosis [153]. There is ongoing work in this area, including elucidating the modulatory effects of liraglutide on the EAT microenvironment (NCT03260881) (Table 3). 

### 6.7. Remodeling Plaque towards Stability 

Plaques that are vulnerable to rupture have a distinctive pathological phenotype, comprising extensive inflammatory infiltration and a necrotic core with an overlying thin fibrous cap [154]. Matrix metalloproteinases (MMPs) degrade the collagen and other extracellular matrix proteins in thin-cap fibroatheromas, resulting in increased vulnerability of the fibrous cap to mechanical forces in the artery and the potential for plaque rupture and thrombosis [154]. Exenatide was shown in a murine model of atherosclerosis to increase the collagen content of plaque and decrease MMP-9/MMP-2 activity, thereby helping to stabilise plaque [155]. This has been proposed to occur via activation of tissue inhibitors of metalloproteinases (TIMPs), which are natural inhibitors of MMPs [156]. In another study, semaglutide reduced the plaque content of CD163^+^ macrophages [108], which is associated with decreased intraplaque angiogenesis and a lower risk of plaque haemorrhage and rupture [108,157]. The ability of GLP-1 RAs to passivate or stabilise plaque requires further evaluation in human studies but may be a novel and important mechanism by which this class confers a reduced risk of future and recurrent ischaemic events, including both MI and ischaemic stroke.

### 6.8. Inhibition of Platelet Aggregation and Thrombosis 

Platelet aggregation following plaque rupture or erosion causes arterial thrombosis, which restricts tissue perfusion and leads to infarction distal to the occlusion site. In addition to potentially making plaques less vulnerable to rupture, there is also some evidence that GLP-1 RAs may exert anti-thrombotic properties. In human cellular studies, exenatide inhibited the aggregation of platelets induced by thrombin, adenosine diphosphate (ADP) and collagen [158]. Meanwhile, low-dose liraglutide acutely attenuated the aggregation of platelets induced by thromboxane in a small study of nine participants with obesity and prediabetes [159]. Both GLP-1 amides and liraglutide have been demonstrated to impart numerous other effects on platelet biology; these include reduction of arachidonic-induced oxidative stress, increased cGMP/protein kinase G (PKG)/vasodilator-stimulated phosphoprotein (VASP) signalling and reduced PI3K/PKB and MAPK signalling [160]. Furthermore, GLP-1R agonism may also inhibit platelet activation by causing platelets to have higher levels of NO and lower P-selectin and platelet activation complex-1 (PAC-1) [161].

Collectively, there is therefore a large body of mechanistic data to show that GLP-1 RAs exert pleiotropic actions on platelets and immune and vascular cells that would protect against the development and complications of atherosclerosis independently of, and in addition to, their canonical effects on glycaemia, appetite and weight regulation. 

## 7. Future Directions 

Although the striking cardiometabolic benefits of GLP-1 RAs in T2DM are well-established, there is evidence that prescribing rates around the world have been suboptimal. Alarmingly, this has also applied to specialist clinic settings for those with T2DM and established CAD, including prior MI [162]. This clinical inertia may stem from insufficient awareness among cardiologists and other physicians regarding the cardioprotective nature of GLP-1 RAs and the mechanistic uncertainty about how they exert their benefits. Paradoxically, as the weight loss properties of GLP-1 RAs have gained widespread attention through traditional and social media platforms, the increasing demand for this off-label indication has created a global shortage, limiting accessibility in many countries. This has prompted critical discussions about prioritising access between different patient subgroups. For many patients with T2DM who have failed other treatments, adequate glycaemic control may be reliant on GLP-1 RA use. It is simultaneously true that with obesity disproportionately affecting lower socioeconomic populations [163], designating weight loss as an off-label indication for GLP-1 RAs could be tantamount to financial gatekeeping, potentially further entrenching health inequities within these populations. Similarly, as we learn more about how GLP-1 RAs protect against atherosclerosis and CVD with trials such as SELECT [91], we will need to consider how management of ASCVD with these agents fits within this wider issue of equitable access and the effects of withdrawal of treatments, which can result in weight regain and reversal of cardiometabolic benefits [164]. 

Opportunities for intensifying cardiovascular risk reduction in patients with T2DM have also been generated by combining GLP-1 RAs with other therapies, including SGLT2 inhibitors [165]. Based on their potential synergism, a fusion therapy comprising GLP-1 RA with the potent cholesterol-lowering of proprotein convertase subtilisin/kexin type 9 inhibition (PCSK9i) is being developed, especially with an eye to patients who are intolerant or refractory to statins [166]. Recent trial evidence also suggests a beneficial effect of GLP-1 RA use on diabetic renal disease [167], which supports signals observed in the early GLP-1 trials [9].

Beyond GLP-1 RAs, a growing wave of more potent incretin-based treatments now offers the potential for even better glycaemic control and weight loss benefits. Tirzepatide, a novel ‘twincretin’ targeting both GLP-1 and GIP receptors, has demonstrated efficacy for treating T2DM in the SURPASS clinical trial program [168] and overweight/obesity in the SURMOUNT studies [169]. This resulted in FDA approval for T2DM in May 2022 and chronic weight management in late 2023. Although the cardiovascular safety of tirzepatide has been established in short-term trials [170], proof of beneficial effects on hard cardiovascular outcomes is still awaited with dedicated outcome trials ongoing (SURPASS-CVOT, NCT04255433; SURMOUNT-MMO, NCT05556512) (Table 3). Additionally, within the incretin drug pipeline, retatrutide, a unimolecular peptide with tri-agonist activity at the GIP, GLP-1 and glucagon receptors, recently showed promising results in a phase 2 evaluation for both weight loss and glycaemic control [171]. Finally, orforglipron is being developed as the first nonpeptide oral alternative to injectable GLP-1 RAs to help overcome adherence issues of injectable therapies. Early trials have found benefits for both obesity [172] and diabetes [173], expanding oral GLP-1 RA choices. Both retatrutide and orforglipron are now undergoing phase 3 evaluation (Table 3). The increased availability of oral options for GLP-1 RAs may also open up the possibility of their incorporation into fixed-dose polypill combinations [174]. This is particularly important as polypill fixed-dose combinations with statins, ACE inhibitors and aspirin have previously been shown to increase adherence and reduce cardiovascular endpoints by 24–30% compared to standard care [175].

## 8. Conclusions 

As the field of anti-diabetic treatment continues to evolve rapidly, GLP-1 RAs are gaining attention for their beneficial effects on cardiovascular outcomes and specifically their ability to lower the risk of ischaemic events. A growing body of evidence indicates that beyond improving cardiometabolic health, these drugs may also exert direct anti-atherosclerotic properties via pleiotropic mechanisms that target numerous cellular and molecular mediators implicated in the different stages of plaque formation, destabilisation and thrombosis. Given the established beneficial effects on ASCVD risk reduction, GLP-1 RAs should be considered as the preferred agent for decreasing ASCVD risk in patients with T2DM and overweight/obesity.

## Figures and Tables

**Table 1:** Chemical and pharmacokinetic properties of GLP-1 RAs. Adapted from Nauck and Meier, 2019; Tahrani, Barnett and Bailey, 2016; Yoon et al., 2020 [42,43,44].

Agent Name	Brand Name	Molecular Structure	Molecular Weight (g/mol)	Administration	Dosing	Half-Life (t_1/2_)	T_max_
Exenatide	Byetta^®^	Modelled on exendin-4 from lizard *Heloderma suspectum* 39 a.a. peptide amide	4186.6	S/C twice daily. 1 h before meals, >6 h apart Uptitration recommended	5 µg 10 µg	3–4 h	2 h
Exenatide Extended-Release	Bydureon Bcise^®^	Modelled on exendin-4 39 a.a. synthetic peptide, suspension of microspheres in a MCT vehicle	4186.6	S/C once weekly Independent of meals	2 mg	3–4 h §	6 w
Lixisenatide †	Adlyxinc^®^	Modelled on exendin-4 44 a.a., amidated at the C-terminal a.a. Contains poly-lysine tail	4858.6	S/C once daily 1 h before first meal of day Uptitration recommended	10 µg 20 µg	2–4 h	1–2 h
Liraglutide	Victoza^®^	Modelled on mammalian GLP-1 Substitution of arginine for lysine at position 34. Attachment of a C-16 fatty acid (palmitic acid) at position 26	3751.3	S/C once daily Independent of meals Uptitration recommended	0.6 mg 1.2 mg 1.8 mg	10–15 h	9–12 h
Semaglutide (S/C)	Ozempic^®^	Modelled on mammalian GLP-1 Albumin binding, with a hydrophilic spacer and a C18 fatty di-acid	4113.6	S/C once weekly Independent of meals Uptitration recommended	0.5 mg 1 mg 2 mg	1 w	1–3 d
Semaglutide (Oral)	Rybelsus^®^	Modelled on mammalian GLP-1 Albumin binding, with a hydrophilic spacer and a C18 fatty di-acid SNAC for enhanced stomach absorption	4113.6	Oral once daily >30 min before first meal of day Uptitration recommended	7 mg 14 mg	1 w	1 h
Dulaglutide	Trulicity^®^	Modelled on mammalian GLP-1. Fusion of 2 disulfide-linked GLP-1 chains to the Fc region of human IgG4	59,669.8	S/C once weekly Independent of meals	0.75 mg 1.5 mg	4 d	12–72 h
Albiglutide	Tanzeum^®^	Modelled on mammalian GLP-1 2 chains of GLP-1 bound to albumin	72,070.4	S/C once weekly Independent of meals Uptitration recommended	30 mg 50 mg	6–7 d	3–5 d
Efpeglenatide ††	FDA approval pending	Exendin-based Conjugation of exendin chain to IgG4 Fc fragment	NA	S/C once weekly Dosing time/regime not yet standardised	4 mg 6 mg	135–180 h	72–144 h

§ Not formally assessed. However, given that this drug works by having exenatide bound to microspheres, it technically has the same half-life as the short-acting version, and this results in significant interindividual variability in absorption. † Lixisenatide has been discontinued by Sanofi in the UK and the USA. †† Since FDA approval is pending for efpeglenatide, standardised dosing and its brand name are not available. Abbreviations: a.a.: amino acid, d: days, h: hours, MCT: medium-triglyceride, S/C: subcutaneous, SNAC: sodium N-[8-(2-hydroxybenzoyl) amino caprylate, T_max_: time to peak drug concentration, w: weeks.

**Table 2:** Review of Cardiovascular Outcome Trials (CVOTs) for GLP-1 Receptor Agonists.

Trial	ELIXA	LEADER	SUSTAIN-6	EXSCEL	Harmony Outcomes	REWIND	PIONEER 6	AMPLITUDE-O
Reference	Pfeffer et al. (2015) [53]	Marso et al. (2016) [54]	Marso et al. (2016) [50]	Holman et al. (2017) [55]	Hernandez et al. (2018) [56]	Gerstein et al. (2019) [57]	Husain et al. (2019) [58]	Gerstein et al. (2021) [59]
Agent Name	Lixisenatide	Liraglutide	Semaglutide (S/C)	Exenatide	Albiglutide	Dulaglutide	Semaglutide (Oral)	Efpeglenatide
Active Intervention	Daily S/C lixisenatide (10–20 μg)	Daily S/C liraglutide (1.8 mg)	Weekly semaglutide S/C (0.5 or 1.0 mg)	Weekly S/C ER exenatide (2 mg)	Weekly S/C albiglutide (30–50 mg)	Weekly S/C dulaglutide (1.5 mg)	Daily oral semaglutide (14 mg)	Weekly S/C efpeglenatide (4 or 6 mg)
Participant Number	6068	9340	3297	14,752	9463	9901	3183	4076
Patient Cohort Characteristics	T2DM + ACS in last 180 d Age ≥ 30	T2DM + high CV risk (age ≥ 50 with prior CAD, stroke, PVD, HF, CKD; or age ≥ 60 with CV RFs)	T2DM + high CV risk (prior CAD, stroke, PVD, HF, CKD; or age ≥ 60 with CV RFs)	T2DM +/− prior CVD (70% had prior CVD)	T2DM + age ≥ 40 + prior CVD, stroke, PAD	T2DM + age ≥ 50 + CVD or CV RFs	High CV risk (with established CVD or CKD; or age ≥ 60 with CV RFs)	T2DM + age ≥ 18 + CVD; or T2DM + age ≥ 50 + CV RFs + CKD
Median Follow-Up	2.1 years	3.8 years	2 years	3.2 years	1.5 years	5.4 years	1.3 years	1.8 years
Changes in *HbA1c*	−0.27%	−0.40%	−1.1% (0.5 mg) −1.4% (1.0 mg)	−0.7%	−0.63% (8 m) −0.52% (16 m)	−0.61%	−0.7%	−1.24%
Definition of Primary Endpoint	CV death, MI, stroke or hospitalisation for unstable angina	First occurrence CV death, nonfatal MI or stroke	First occurrence CV death, nonfatal MI or stroke	First occurrence CV death, nonfatal MI or stroke	First occurrence CV death, nonfatal MI or stroke	First occurrence CV death, nonfatal MI or stroke	First occurrence CV death, nonfatal MI or stroke	First occurrence death from CV or undetermined causes, nonfatal MI or stroke
MACE Result (HR)	1.02 (95% CI [0.89, 1.17], *p* = 0.81)	0.87 (95% CI [0.78, 0.97], *p* = 0.01)	0.74 (95% CI [0.58, 0.95], *p* = 0.02)	0.91 (95% CI [0.83, 1.00], *p* = 0.06)	0.78 (95% CI [0.68–0.90], *p* = 0.0006)	0.88 (95% CI [0.79–0.99], *p* = 0.026)	0.79 (95% CI [0.57, 1.11], *p* = 0.001)	0.73 (95% CI [0.58–0.92], *p* = 0.007)
All-Cause Death (HR)	0.94 (95% CI [0.78–1.13])	0.85 (95% CI [0.74–0.97])	1.05 (95% CI [0.74–1.50])	0.86 (95% CI [0.77–0.97])	0.95 (95% CI [0.79–1.16])	0.90 (95% CI [0.80–1.01])	0.51 (95% CI [0.31–0.84])	0.78 (95% CI [0.58–1.06])
Cardiovascular Death (HR)	0.98 (95% CI [0.78–1.22])	0.78 (95% CI [0.66–0.93])	0.98 (95% CI [0.65–1.48])	0.88 (95% CI [0.76–1.02])	0.93 (95% CI [0.73–1.19])	0.91 (95% CI [0.78–1.06])	0.49 (95% CI [0.27–0.92])	0.72 (95% CI [0.50–1.03])
Nonfatal Myocardial Infarction (HR)	1.03 (95% CI [0.87–1.22]) *	0.88 (95% CI [0.75–1.03])	0.74 (95% CI [0.51–1.08])	0.97 (95% CI [0.85–1.10]) *	0.75 (95% CI [0.61–0.90]) *	0.96 (95% CI [0.79–1.16])	1.18 (95% CI [0.73–1.90])	0.78 (95% CI [0.55–1.10])
Nonfatal Stroke (HR)	1.12 (95% CI [0.79–1.58]) **	0.89 (95% CI [0.72–1.11])	0.61 (95% CI [0.38–0.99])	0.85 (95% CI [0.70–1.03]) **	0.86 (95% CI [0.66–1.14]) **	0.76 (95% CI [0.61–0.95])	0.74 (95% CI [0.35–1.57])	0.80 (95% CI [0.48–1.31])
Hospitalisation for Heart Failure (HR)	0.96 (95% CI [0.75–1.23])	0.87 (95% CI [0.73–1.05])	1.11 (95% CI [0.77–1.61])	0.94 (95% CI [0.78–1.13])	0.85 (95% CI [0.70–1.04]) ***	0.93 (95% CI [0.77–1.12])	0.86 (95% CI [0.49–1.55])	0.61 (95% CI [0.38–0.98])
Effect on Heart Rate	+0.4 bpm	+3.0 bpm	+2.1 bpm (0.5 mg) +2.4 bpm (1.0 mg)	+2.51 bpm	+1.3 bpm	+1.87 bpm	+4 bpm	+3.9 bpm
Effect on Weight from Baseline	−0.6 kg	−2.3 kg	−3.6 kg (0.5 mg) −4.9 kg (1.0 mg)	−1.27kg	−0.66 kg (8 m) −0.83 kg (16 m)	−1.46 kg	−4.2kg	−2.6 kg
Effect on Systolic Blood Pressure	−0.8 mmHg	−1.2 mmHg	−3.4 mmHg (0.5 mg) −5.4 mmHg (1.0 mg)	−1.57 mmHg	−0.65 mmHg (8 m) −0.67 mmHg (16 m)	−1.70 mmHg	−2.6 mmHg	−1.5 mmHg
Overall Effect on CVD	No benefit	MACE benefit + reduced all-cause and CV mortality	MACE benefit + reduced stroke	No MACE benefit but reduced all-cause mortality	MACE benefit + reduced MI	MACE benefit + reduced stroke	No MACE benefit but reduced all-cause and CV mortality	MACE benefit + reduced HF hospitalisation

* This was reported as an aggregate of fatal and nonfatal myocardial infarction. ** This was reported as an aggregate of fatal and nonfatal stroke. *** This was reported as a composite of death from cardiovascular causes and hospital admission for heart failure. Trial abbreviations: AMPLITUDE-O: Effect of Efpeglenatide on Cardiovascular Outcomes; ELIXA: Evaluation of Lixisenatide in Acute Coronary Syndrome; EXSCEL: Exenatide Study of Cardiovascular Event Lowering; Harmony Outcomes: Effect of Albiglutide, When Added to Standard Blood Glucose Lowering Therapies, on Major Cardiovascular Events in Subjects With Type 2 Diabetes Mellitus; LEADER: Liraglutide Effect and Action in Diabetes: Evaluation of Cardiovascular Outcome Results; PIONEER 6: Peptide Innovation for Early Diabetes Treatment 6; REWIND: Researching cardiovascular Events with a Weekly INcretin in Diabetes; SUSTAIN-6: Trial to Evaluate Cardiovascular and Other Long-term Outcomes with Semaglutide in Subjects with Type 2 Diabetes. Other abbreviations: ACS: acute coronary syndrome; bpm: beats per minute; CAD: coronary artery disease; CI: confidence interval; CKD: chronic kidney disease; CV: cardiovascular; CVD: cardiovascular disease; ER: extended release; HbA1c: glycated hemoglobin; HF: heart failure; HR: hazard ratio; MACE: major adverse cardiovascular event; MI: myocardial infarction; m: months; S/C: subcutaneous; T2DM: type 2 diabetes mellitus; PAD: peripheral arterial disease; PVD: peripheral vascular disease; RFs: risk factors.

**Table 3:** Active, In-Progress and Upcoming GLP-1 RA/incretin clinical trials registered on www.clinicaltrials.gov.

Trial Name	ClinicalTrials.gov ID	Purpose	Phase	Trial Status	Estimated Completion	Expected Sample Size	Inclusion Criteria	Active Intervention	Primary Outcome Measures
LEADPACE	NCT04146155	Liraglutide in diabetic patients with PAD	Phase 4	Unknown	December 2021	200	Age > 40; T2DM; HbA1c: 7.5–14%; PAD	Liraglutide	Initial and absolute claudication distance and assessment of limb ischaemia
STARDUST	NCT04881110	Liraglutide on peripheral arterial perfusion	Phase 4	Unknown	June 2022	50	T2DM; HbA1c 6.5–8%; PAD	Liraglutide	Transcutaneous oxygen pressure (mmHg) on anterior and posterior tibial arteries
STOP	NCT03985384	Semaglutide on coronary plaque progression and composition using serial CTCA	Phase 4	Completed, awaiting publication	December 2022	140	Age ≥ 40; T2DM; HbA1c ≥ 7.0%; prior ASCVD or ≥1 CV RF	Semaglutide S/C	Change in noncalcified plaque volume on CTCA
SAMAS	NCT05147896	Anti-atherosclerotic mechanisms of oral semaglutide	Phase 4	Active, recruiting	December 2023	100	T2DM; HbA1c ≤ 8.5% BMI ≥ 30	Semaglutide oral	cIMT; endothelial function; arterial stiffness
Effects of Liraglutide on Epicardial Fat Pro-Inflammatory Genes in Type 2 Diabetes and Coronary Artery Disease	NCT03260881	Liraglutide on the inflammatory environment of EAT	Phase 4	Active, recruiting	December 2023	40	T2DM; HbA1c ≤ 9%; BMI ≥ 27 and/or WC ≥ 102 cm (men) or 88 cm (women); stable CAD requiring CABG	Liraglutide for minimum of 4–12 w prior to CABG	EAT inflammation as measured by mRNA and protein expression of tumour necrosis factor (TNF)-alpha and interleukin (IL)-6 from blood sample
STRIDE	NCT04560998	Semaglutide on walking distance in PAD + T2DM	Phase 3	Active, not recruiting	July 2024	800	T2DM; PAD	Semaglutide S/C	Change in maximum walking distance
SOUL	NCT03914326	Oral semaglutide in T2DM with ASCVD and/or CKD	Phase 3	Active, not recruiting	July 2024	9642	Age ≥ 50; T2DM; HbA1c 6.5–10.0%; CAD, CeVD, PAD or CKD	Semaglutide oral	Time to first CV death, nonfatal MI or stroke
SURPASS-CVOT	NCT04255433	Tirzepatide vs. dulaglutide in T2DM and high CV risk	Phase 3	Active, not recruiting	October 2024	13,299	T2DM; HbA1c 7.0–10.5%; ASCVD; BMI ≥ 25	Tirzepatide S/C	Time to first CV death, MI or stroke
SURMOUNT-MMO	NCT05556512	Tirzepatide vs. placebo in nondiabetic overweight/obesity with high ASCVD risk	Phase 3	Active, recruiting	October 2027	15,000	Age ≥ 40; BMI ≥ 27; prior ASCVD or high CV risk	Tirzepatide S/C	Time to first all-cause death, nonfatal MI, nonfatal stroke, coronary revascularisation or heart failure events
TRIUMPH-3	NCT05882045	Retatrutide vs. placebo in obesity with prior CVD	Phase 3	Active, recruiting	February 2026	1800	BMI ≥ 35.0; prior MI, stroke or PAD	Retatrutide S/C	Percent change from baseline in body weight
ACHIEVE-4	NCT05803421	Orforglipron vs. insulin glargine in T2DM and overweight/obesity and high CV risk	Phase 3	Active, recruiting	December 2025	2620	T2DM; HbA1c 7.0–10.5%; BMI ≥ 25; high CV risk	Orforglipron oral	Time to first CV death, MI, stroke or hospitalisation for unstable angina

Trial abbreviations: LEADPACE: Liraglutide Efficacy and Action on Type 2 Diabetes With Peripheral Atherosclerotic intErmittent Claudication: a Prospective, 24-week, Multicenter, Randomized, Controlled Clinical Study; STARDUST: Effects of the Glucagon Like-peptide 1 (GLP-1) Receptor Agonist Liraglutide on Lower Limb Perfusion in People With Type 2 Diabetes and Peripheral Artery Disease: a Randomized Controlled Trial; STOP: Semaglutide Treatment on Coronary Plaque Progression Trial; SAMAS: Semaglutide Anti-Atherosclerotic Mechanisms of Action Study in Type 2 Diabetes Patients; STRIDE: Effects of Semaglutide on Functional Capacity in Patients With Type 2 Diabetes and Peripheral Arterial Disease; SOUL: Semaglutide Cardiovascular Outcomes Trial in Patients With Type 2 Diabetes; SURPASS-CVOT: The Effect of Tirzepatide Versus Dulaglutide on Major Adverse Cardiovascular Events in Patients With Type 2 Diabetes; SURMOUNT-MMO: A Phase 3, Randomized, Double-blind, Placebo-Controlled Study to Investigate the Effect of Tirzepatide on the Reduction of Morbidity and Mortality in Adults With Obesity; TRIUMPH-3: A Study of Retatrutide (LY3437943) in Participants With Obesity and Cardiovascular Disease; ACHIEVE-4: A Study of Daily Oral Orforglipron (LY3502970) Compared With Insulin Glargine in Participants With Type 2 Diabetes and Obesity or Overweight at Increased Cardiovascular Risk. Other abbreviations: ASCVD: atherosclerotic cardiovascular disease; BMI: body mass index; CAD: coronary artery disease; CABG: coronary artery bypass graft; CeVD: cerebrovascular disease; cIMT: carotid intima–media thickness; CKD: chronic kidney disease; CTCA: computed tomography coronary angiogram; CV: cardiovascular; CVD: cardiovascular disease; EAT: epicardial adipose tissue; HbA1c: glycated hemoglobin; MI: myocardial infarction; PAD: peripheral artery disease; RF: risk factor; S/C: subcutaneous; T2DM: type 2 diabetes mellitus; WC: waist circumference.
